# Supplementary material for: The Effect of the COVID-19 Pandemic on Outpatient Antibiotic Prescription Rates in Children and Adolescents—A Claims-Based Study in Germany
Source: Antibiotics (Basel). 2022 Oct 18;11(10):1433. doi: 10.3390/antibiotics11101433 (PMC9598667; doi:10.3390/antibiotics11101433)
Supplement: Supplementary file 1 [file antibiotics-11-01433-s001.zip › antibiotics-1973161-supplementary.pdf]

Supplementary Materials

Table S1. The size of the total and study population in the age segment 0–14 years and annual prescription rate in Germany in the period 2010 to 2021

Year	Total population[§]	Study population	
	**N**	**N (%)**	**Prescription rate per 1,000 children**
2010	10,941,201	9,132,132 (83.5)	731
2011	10,773,006	8,998,191 (83.5)	671
2012	10,682,903	8,880,007 (83.1)	617
2013	10,642,432	8,808,031 (82.8)	623
2014	10,686,723	8,805,108 (82.4)	559
2015	10,881,126	8,872,685 (81.5)	509
2016	11,048,568	9,016,718 (81.6)	516
2017	11,171,759	9,264,105 (82.9)	455
2018	11,290,815	9,389,190 (83.2)	428
2019	11,391,259	9,500,009 (83.4)	401
2020	11,477,800	9,619,883 (83.8)	227
2021	11,606,935	9,688,483 (83.5)	188

[§] Data for the total number of children and adolescents in the age segment 0–14 years in Germany originate from the Federal Statistical Office (https://www.destatis.de)

Table S2. The size of the total and study population in the age segment 0–14 years and annual prescription rate in 2021 by age group

	**Total population[§]**		**Study population**		
**Age group in years**	N	%	N	%	Prescription rate per 1,000 children
0–1	1,572,049	13.5	1,309,696	13.5	215
2–5	3,219,426	27.7	2,702,301	27.9	319
6–9	3,071,999	26.5	2,531,550	26.1	140
10–14	3,743,461	32.3	3,144,936	32.5	103

[§] Data for the total number of children and adolescents in the age segment 0–14 years in Germany originate from the Federal Statistical Office (https://www.destatis.de).

Table S3. Monthly antibiotic prescription rate per 1,000 children aged between 0 and 14 years by antibiotic subgroup in the period 2019 to 2021

**Antibiotic subgroup**	**2019**												**2020**												**2021**											
	**1**	**2**	**3**	**4**	**5**	**6**	**7**	**8**	**9**	**10**	**11**	**12**	**1**	**2**	**3**	**4**	**5**	**6**	**7**	**8**	**9**	**10**	**11**	**12**	**1**	**2**	**3**	**4**	**5**	**6**	**7**	**8**	**9**	**10**	**11**	**12**
narrow-spectrum penicillins	7.6	8.2	8.1	7.3	7.2	5.3	4.9	3.1	4.3	4.8	6.2	7.3	7.3	7.6	5.8	1.3	1.0	1.2	1.3	1.3	1.6	1.6	1.7	1.5	0.9	0.7	1.0	0.8	0.9	1.2	1.5	1.4	2.0	2.2	2.3	2.0
extended-spectrum penicillins	13.7	17.6	13.1	10.4	8.3	6.4	6.4	4.2	6.5	7.8	9.8	12.3	12.9	16.5	11.0	1.9	1.7	2.3	3.4	3.6	4.1	3.5	3.2	2.8	1.4	1.2	2.0	1.9	2.2	4.0	6.1	4.7	8.0	13.2	12.3	8.3
penicillins with ß-lactamase inhibitor	1.6	2.0	1.7	1.5	1.4	1.3	1.5	1.2	1.3	1.3	1.4	1.7	1.7	2.1	1.8	0.7	0.7	0.9	1.0	1.1	1.1	0.9	0.9	0.8	0.6	0.6	0.7	0.7	0.7	1.2	1.4	1.2	1.5	1.8	1.7	1.3
first-generation cephalosporins	0.6	0.7	0.7	0.6	0.6	0.4	0.4	0.3	0.4	0.4	0.5	0.5	0.6	0.6	0.5	0.1	0.1	0.1	0.1	0.1	0.2	0.1	0.2	0.1	0.1	0.1	0.1	0.1	0.1	0.1	0.2	0.1	0.2	0.2	0.2	0.2
second-generation cephalosporins	10.9	12.9	10.6	9.1	8.0	6.8	7.0	5.1	6.5	7.0	8.0	9.2	9.4	10.8	8.1	2.5	2.2	2.6	3.3	3.5	3.9	3.2	3.2	2.9	1.9	1.7	2.3	2.0	2.0	3.3	4.3	3.4	4.8	6.4	6.2	4.7
third-generation cephalosporins	2.1	2.6	2.2	1.8	1.6	1.2	1.3	1.0	1.2	1.4	1.7	2.0	2.0	2.3	1.9	0.6	0.6	0.6	0.8	0.7	1.0	0.9	0.9	0.8	0.6	0.5	0.7	0.6	0.6	0.9	1.1	0.9	1.2	1.7	1.6	1.2
macrolides	5.0	6.3	4.5	3.6	2.9	2.2	2.2	1.6	2.7	2.9	4.1	4.5	4.6	5.5	4.0	0.8	0.5	0.6	0.9	1.0	1.6	1.2	1.1	0.9	0.5	0.4	0.7	0.6	0.6	1.1	1.8	1.2	2.2	3.3	3.0	1.9
sulfonamides/trimethoprim	1.5	1.4	1.3	1.2	1.3	1.0	1.2	1.1	1.3	1.3	1.5	1.4	1.5	1.3	1.2	0.8	0.8	0.9	1.0	0.9	1.1	1.2	1.2	1.2	1.0	1.0	1.2	1.0	1.0	1.1	1.1	1.0	1.2	1.2	1.3	1.2
other antibiotics	0.6	0.6	0.6	0.6	0.6	0.5	0.6	0.5	0.7	0.7	0.7	0.7	0.6	0.5	0.6	0.4	0.4	0.5	0.5	0.5	0.6	0.6	0.6	0.5	0.4	0.4	0.4	0.4	0.4	0.5	0.5	0.5	0.6	0.6	0.6	0.5

See Figure 2 for grafical presentation.
